# Smartphone based optical spectrometer for diffusive reflectance spectroscopic measurement of hemoglobin

**DOI:** 10.1038/s41598-017-12482-5

**Published:** 2017-09-22

**Authors:** Perry Edwards, Chenji Zhang, Baigang Zhang, Xiangqian Hong, Vivek K. Nagarajan, Bing Yu, Zhiwen Liu

**Affiliations:** 1 Atoptix, Inc., 200 Innovation Blvd., Suite, 234–1, 16803 State College, PA USA; 2 Department of Electrical Engineering and Materials Research Institute, The Pennsylvania State University, University Park, 16802 PA USA; 3 Department of Biomedical Engineering, Marquette University and Medical College of Wisconsin, Milwaukee, WI 53233 United States; 4 Department of Biomedical Engineering, The University of Akron, Akron, OH 44325 United States

## Abstract

We report a miniature, visible to near infrared G-Fresnel spectrometer that contains a complete spectrograph system, including the detection hardware and connects with a smartphone through a microUSB port for operational control. The smartphone spectrometer is able to achieve a resolution of ~5 nm in a wavelength range from 400 nm to 1000 nm. We further developed a diffuse reflectance spectroscopy system using the smartphone spectrometer and demonstrated the capability of hemoglobin measurement. Proof of concept studies of tissue phantoms yielded a mean error of 9.2% on hemoglobin concentration measurement, comparable to that obtained with a commercial benchtop spectrometer. The smartphone G-Fresnel spectrometer and the diffuse reflectance spectroscopy system can potentially enable new point-of-care opportunities, such as cancer screening.

## Introduction

Hemoglobin is an important biomarker for diagnosing a variety of clinical conditions^1–8^. For instance, angiogenesis and hypoxia are two salient features of tumor growth. Thus, non-invasive measurement of both oxy- and deoxy-hemoglobin in tissue, from which the total hemoglobin concentration and its oxygenation state can be determined, is a promising approach for early detection and screening of breast cancer^9–11^, oral cancer^12–15^, cervical cancer^16–18^, and many other malignancies. Therefore, there is an important need for developing a portable and cost-efficient tool to perform non-invasive measurement of hemoglobin at local tissues for point of care (POC) applications. Note that current gold standard for diagnosing these conditions is biopsy, which is invasive, costly, and not easily accessible in resource scarce communities and developing regions.

Diffuse reflectance spectroscopy (DRS) is a well-established method to measure hemoglobin in tissue.^12,13,19–23^ Unlike specular reflection, in DRS light penetrates deep into a tissue and re-emerges to the surface only after undergoing interactions with the tissue, such as absorption by chromophores and multiple scatterings by cellular and intercellular structures. As such, the diffuse reflectance spectrum contains important information of chromophore concentrations and tissue scattering properties. In the visible wavelength, the most significant absorbers in human tissue are oxy-hemoglobin and deoxy-hemoglobin. Due to its non-invasive nature, DRS can avoid blood drawing and consumables, and thus lends itself to significant cost reduction during usage. Yet, conventional implementation of DRS typically involves the use of bulky and costly optical spectrometers, which remain a significant hindrance for gaining wider accessibility, especially in resource-limited areas.

In recent years, there is an increasing interest in developing portable, low-cost optical spectroscopic devices and integrating them with mobile phones to leverage the ease of accessing and processing data using mobile computing technology.^24–29^ For instance, several smartphone based spectrometers were demonstrated with wavelength resolutions ranging from 2 to 15 nm^25,28,30,31^. In large part due to the competing requirements of size, cost, and performance, it remains challenging to realize a truly miniature, cost-effective, and high-performance mobile phone spectrometer. Developing these capabilities is critical for enabling POC applications that demand high accuracy in measurements at low cost. In the past several years, G-Fresnel, a diffractive optical element with the dual functionality of focusing and dispersion has been developed for implementing miniaturized high-resolution optical spectrometers^32–35^. A transmission G-Fresnel, as used in this work, can be fabricated via soft lithography, by sandwiching polydimethylsiloxane (PDMS) prepolymer between a negative Fresnel lens mold and a grating mold^35^. As a result, the fabricated device consists of a Fresnel lens pattern in one side to focus or collimate impinging light, and a grating pattern on the other side to disperse the different constituent wavelengths. A smartphone spectrometer was recently demonstrated, by directly positioning a G-Fresnel in front of the built-in camera of a smartphone; application to Bradford assay of protein concentration was also studied^36^. Albeit ultralow cost, using the built-in camera as a detector, however, both poses alignment challenges to accommodate the ever-changing positions of the back-camera across different smartphone models, and necessitates workarounds to undo the built-in image processing that often wrecks havoc on spectral images aquired using a smartphone camera. To overcome the challenge, in this work, we developed a G-Fresnel spectrometer that contains a complete spectrograph system, including the detection hardware and control electronics, and can connect with a smartphone through a microUSB port as an add-on device for operational control. We further developed a DRS system based on this smartphone spectrometer and demonstrated quantitive measurement of hemoglobin concentration and reduced scattering coefficient through tissue phantom study. The smartphone spectrometer based DRS system has the potential to be broadly implemented in resource-limited regions.

## Materials and Methods

### Spectrometer design and fabrication

**Figure 1:**
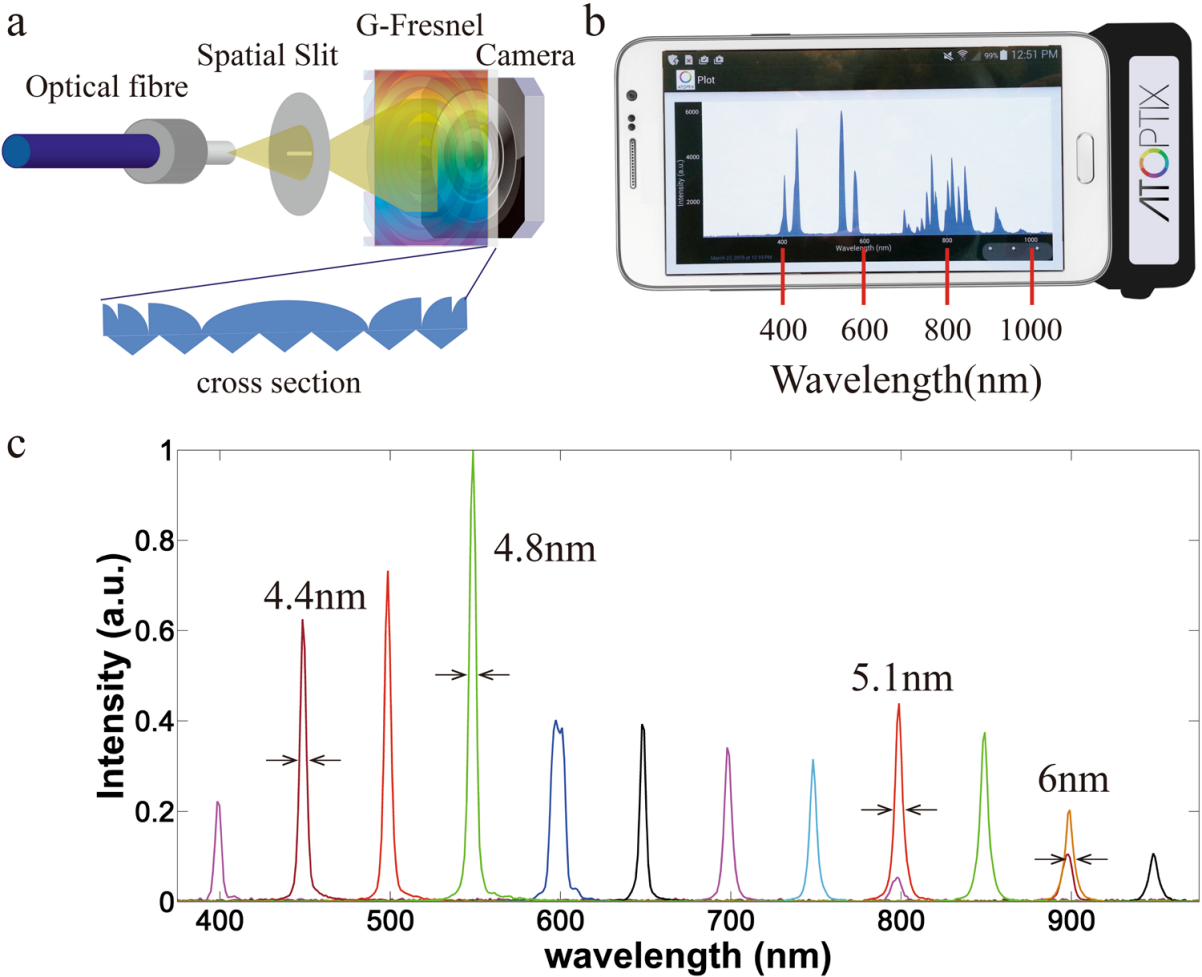
(**a**) A schematic of the G-Fresnel spectrometer; (**b**) a G-Fresnel spectrometer attached to a smartphone displaying the measured spectrum of a calibration lamp; and (**c**) measured spectrum of a tunable narrow-band calibration source showing the wavelength resolution at several representative wavelengths.

A schematic diagram of our G-Fresnel spectrometer is shown in Fig. 1a. An incoming fibre delivers light signal onto a slit. A transmission G-Fresnel device, comprising a grating pattern in one side (600 lines per mm) and Fresnel lens pattern on the other side (focal length: 1 inch at 588 nm), both collimates the light emanating from the slit and disperses the different wavelengths across a mobile camera system containing a built-in lens that focuses the dispersed light onto a complementary metal oxide semiconductor (CMOS) image sensor (Omnivision). The fabrication of G-Fresnel was described in details elsewhere^35^. Briefly, PDMS (Dow Corning Sylgard 184 Silicone) pre-polymer is poured onto the surface of a Fresnel lens and a diffraction grating separately. After baking the PDMS for 12 hours at 60 °C, PDMS is cured completely and a negative Fresnel lens mold and a negative grating mold are made. Afterward, a G-Fresnel can be fabricated by sandwich the PDMS pre-polymer between the two negative molds followed by curing. Raw Bayer pattern images captured by the CMOS detector are first converted to grey level two-dimensional spectral image by summing up the red, green and blue pixel values. The grey level spectral images are then summed by column (along the slit direction) to obtain the final one-dimensional spectral data. The spectrometer enclosure case is fabricated by three-dimensional printing, which has pre-designed slots for positioning and aligning all the components. Slot positions, such as these for holding the input slit, the G-Fresnel, and the camera detection system are first determined on an optical bench. Trial and error is then used to arrive at the final design parameters of the case. Once the case is fabricated, only minor alignment is required. Control electronics is implemented so that the spectrometer can connect to a smartphone using the microUSB charging port, common across Android smartphone platforms. Power is also provided to the CMOS camera from the microUSB connection. An Android APP is developed to configure and control the spectrometer, as well as to transfer the spectral data to the phone for analysis. The Android APP was developed in Eclipse IDE for Java developers. It allows a user to set the integration time, initiate a measurement, store the measured data, display the spectra, and perform simple analysis on the results. Specifically, a spectral capture is initiated by pressing a button on the smartphone screen, which sends a signal to the microelectronics camera controller board over the microUSB to turn on the camera for a desired integration period. The camera then transmits the raw data to the microelectronics controller board, which transmits the data over microUSB back to the APP. The APP stores the data, processes it, and plots the spectrum on the screen. A photo of the smartphone spectrometer is presented in Fig. 1b, which shows the device attached to a smartphone and the APP measurement interface. Wavelength calibration is performed by identifying the wavelength peaks of a calibration lamp and the corresponding pixel positions of the spectral lines captured. The relationship is fitted with a linear curve and the parameters are stored on the phone.

### Diffuse reflectance spectroscopy system

**Figure 2:**
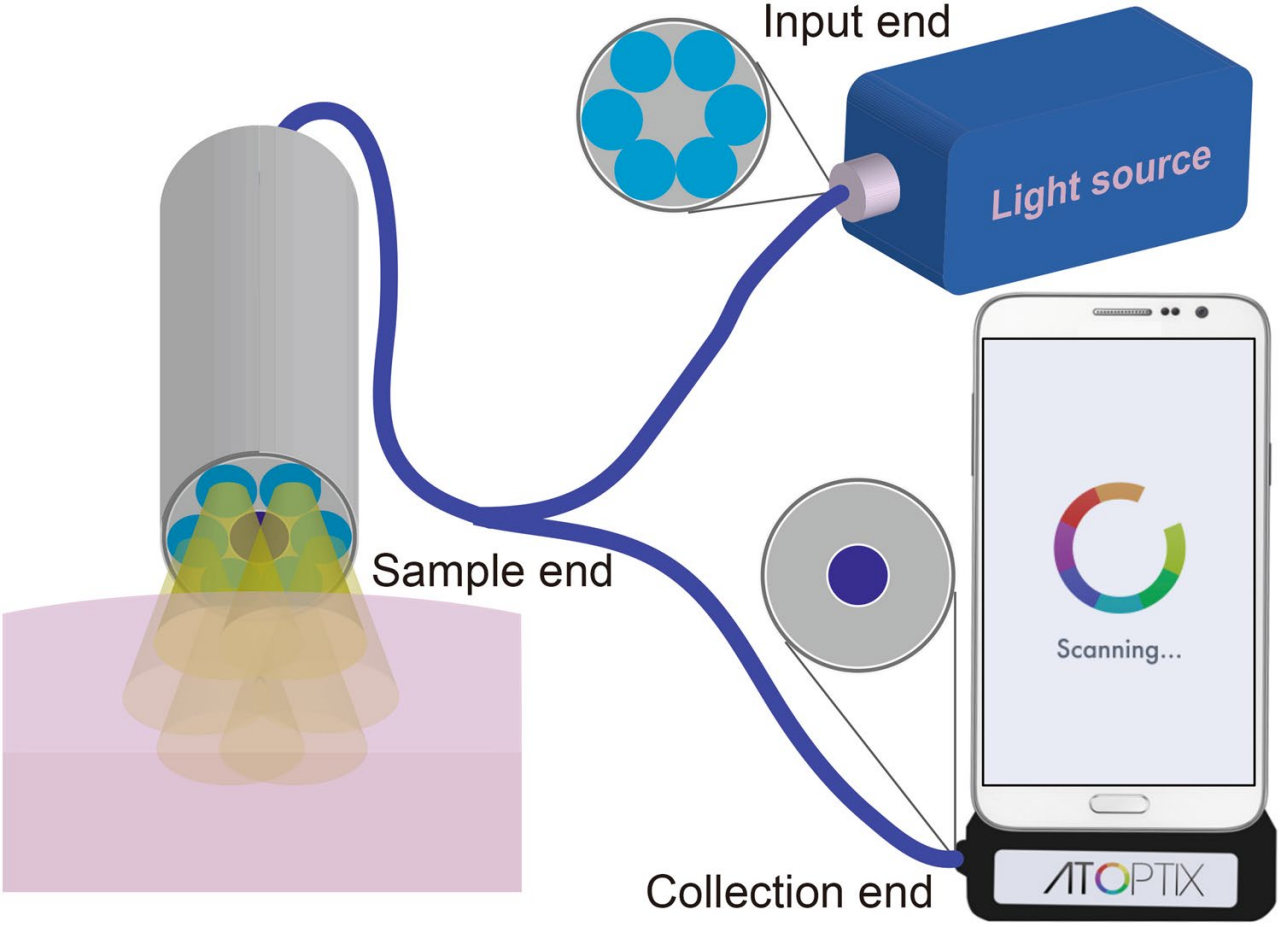
Schematic diagram of the diffuse reflectance spectroscopy experimental system by using the G-Fresnel smartphone spectrometer.

The DRS experimental setup is illustrated in Fig. 2. Light from a broadband tungsten halogen lamp (HL-2000-HP, Ocean Optics) is coupled into an Ocean Optics fibre probe, which consists of 6 multimode fibres surrounding a single fibre in the center (see inset in Fig. 2). The fibre core size is 400 μm, and the diameter of each fibre and the seperation between the centers of neighboring fibres are ~480 μm. The six peripheral fibres are used for illumination while the central fibre collects the diffusely reflected light and delivers it to the G-Fresnel smartphone spectrometer.

### Tissue phantom preparation

The liquid tissue phantom samples are mixtures of human hemoglobin (H0267, Sigma-Aldrich Co. LLC) as the absorber and 1-µm polystyrene microspheres (07310–15, Polysciences, Inc.) as the scatterers dispersed in water. The polystyrene microspheres are used to simulate tissue scattering. Hemoglobin, as the sole absorber in this sample, determines the absorption of the sample following Beer-Lambert Law. Here, we prepared 16 phantoms, which cover a hemoglobin (100% oxidized) concentration range from 5.39 to 36.16 μM in water, by fixing the number of microspheres and titrating the hemoglobin.

### Tissue phantom measurement

In order to measure the diffuse reflectance spectrum, the tip of the fibre probe was brought into contact with the phantom surface and a magnetic stirrer was used to ensure a uniform colloidal suspension of the microspheres throughout the measurements. Individual spectrum was measured from each phantom using the fibre probe and the G-Fresnel smartphone spectrometer with an integration time of 3.6 seconds. Immediately after all phantom measurements, the probe tip was placed in contact with a reflectance standard (Spectralon® SRS-99, LabSphere, Inc.) that has a flat reflectivity across all wavelengths. Experiments were performed at room temperature with ambient light on. Initially, a background measurement was also performed, which accounts the contribution from ambient light. This background spectrum was then subtracted from the measured diffuse reflectance and reference spectra. For comparison, a benchtop DRS system^17^ was also utilized to obtain a diffuse reflectance spectrum from each of the same 16 phantoms.

### Retrieval model

A Monte Carlo (MC) inverse model^37^ of reflectance is used to extract the phantom absorption coefficient µ_a_(λ) and reduced scattering coefficient µ_s_ʹ(λ) as well as hemoglobin concentrations (100% HbO_2_) from the phantom spectrum between 430–630 nm. The wavelength range of 430–630 nm was selected to include the hemoglobin absorption peaks while avoiding those with low signal-to-noise ratio (e.g., between 400–430 nm). In each MC inversion, one of the phantoms was selected as the reference (with known µ_a_(λ), µ_s_ʹ(λ) and reflectance spectrum) to analyze all the other phantoms. This process was repeated 16 times, in which each of the phantoms was used as the reference once. Finally the mean < µ_a_(λ) > and < µ_s_ʹ(λ) > and their standard deviations (or error bars) across the 16 reference phantoms were calculated.

## Results

### Wavelength resolution characterization of the G-Fresnel smartphone spectrometer

The spectral resolution of the G-Fresnel smartphone spectrometer is characterized through a tunable narrow-band (<1 nm) calibration source created by passing a supercontinuum beam through a monochromator (PI Acton SpectraPro). The wavelength is increased at a step of 50 nm. At each wavelength, a spectrum of the calibration source is captured by using the smartphone spectrometer. Results are presented in Fig. 1c. Full-width at half-maximum (FWHM) values are determined at several representative wavelengths and are listed in the figure, indicating a wavelength resolution of about 5 nm. The spectrometer can be used within a wavelength range covering the visible and the near infrared region (~400 nm–1000 nm, limited by the sensitivity of the CMOS detector).

### Tissue phantom experiment

To examin the feasibility of quantitative measurement of hemoglobin concenetration and scattering in tissue, we have used the DRS experimental system to perform proof-of-concept studies on the liquid tissue phantoms.

Two key parameters for DRS, absorption and reduced scattering coefficient, are pre-calculated. The absorption coefficient (µ_a_(λ)) was determined from a spectrophotometer (Lambda 35, PerkinElmer Inc.) measurement of a diluted hemoglobin stock solution and the reduced scattering coefficients (µ_s_ʹ(λ)) was calculated using the Mie’s theory^38^ for known size, density and refractive index of the scatters. The phantom hemoglobin concentrations and the expected µ_a_ and µ_s_ʹ averaged over the wavelength range of 430–630 nm are presented in Table 1.

**Table 1:** Summary of the expected hemoglobin concentrations as well as the mean absorption and scattering coefficients (averaged over 430–630 nm) of the 16 phantoms.

Phantom	Hb concentration (µM)	Expected µ_a_ (cm^−1^)	Expected µʹ_s_ (cm^−1^)
1	5.39	0.46	12.55
2	8.80	0.75	11.95
3	11.90	1.02	11.41
4	14.74	1.26	10.92
5	17.34	1.48	10.46
6	19.73	1.69	10.05
7	21.94	1.87	9.66
8	23.98	2.05	9.31
9	25.89	2.21	8.98
10	27.66	2.36	8.67
11	29.31	2.50	8.38
12	30.86	2.64	8.11
13	32.31	2.76	7.86
14	33.67	2.88	7.62
15	34.95	2.99	7.40
16	36.16	3.09	7.19

**Figure 3:**
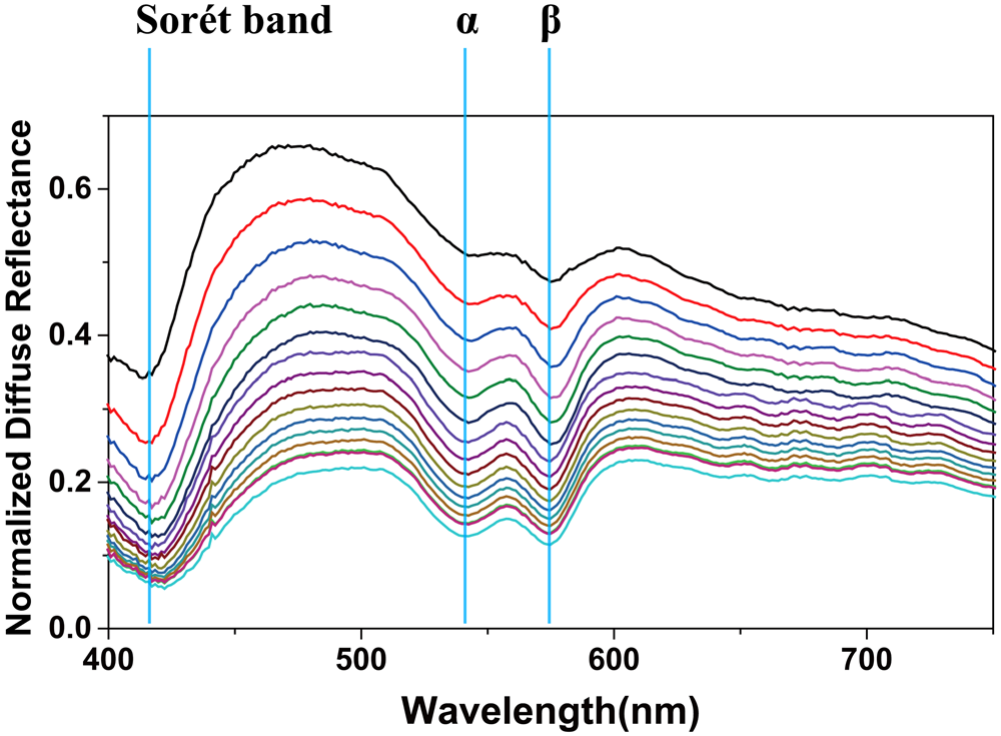
Measured diffuse reflectance spectra at different hemoglobin concentration.

Figure 3 shows the diffuse reflectance spectra measured from all phantoms, obtained by dividing the background subtracted diffuse reflectance spectrum with the background subtracted reference spectrum. The three major absorption bands (Sorét, α and β bands) of oxyhemoglobin are clearly observed. The measured reflectance at the absorption peaks decreases with increasing hemoglobin concentrations as expected.

**Figure 4:**
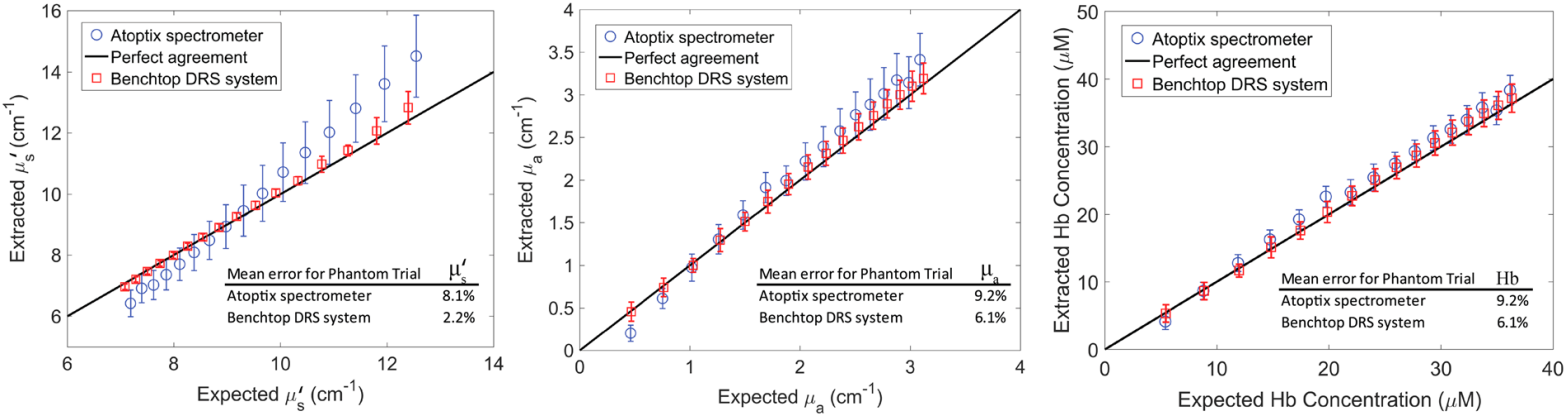
Comparison of retrieved hemoglobin concentration, absorption coefficient and scattering coefficient using the G-Fresnel smartphone spectrometer and these using a benchtop DRS system.

By using the Monte Carlo inversion model, both the absorption and the reduced scattering coefficients were obtained from the diffuse reflectance spectra data measured with the smartphone spectrometer and the benchtop spectrometer. The results are plotted against expected values in Fig. 4a and b. The percent errors, which are the relative difference between the extracted and expected values in µ_a_ and µ_s_ʹ, are also computed and listed in Fig. 4. We have obtained retrieval results with errors of 9.2% and 8.1% for absorption and reduced scattering coefficients, respectively, using the MC retrieval method. From the extracted absorption coefficients, the corresponding hemoglobin concentrations are calculated and presented in Fig. 4c. Typical errors for benchtop visible DRS systems that are used for preclinical and clinical studies are 5–10%^21,39,40^. The error in absorption for this smartphone spectrometer is on the higher end; but it is still within an acceptable range. The extracted µ_a_ and µ_s_’ showed a general trend of higher deviation from the expected values at the two ends, which was due to fewer neighboring phantoms with close µ_a_ and µ_s_’ values used for reference than those in the middle.

Our smartphone spectrometer based measurements compare favorably with the benchtop DRS system developed using a traditional spectrometer (AvaSpec-ULS2048-USB2-RM)^21^, which has errors of 6.1% and 2.2% for retrieved absorption and reduced scattering coefficients, respectively. Note that for tissue studies, only the reference phantom that produces the least error is selected as a reference to extract tissue hemoglobin concentrations.

## Discussion

Smartphone based spectrometers have in recent years attracted significant interests thanks to the ubiquitousness of smartphones and the proven capability of optical spectroscopy for molecular sensing. The use of the G-Fresnel device, with dual-functionalities of focusing/collimation and wavelength dispersion, can significantly simplify the design, reduce the cost, weight and size, while maintains the high performance of optical spectrometers. Our development of an “add-on” spectrometer system attachable to a smartphone leverages the same low-cost and high-performance mobile CMOS camera technology, while allowing for direct access to raw spectral data as opposed to processed ones from a built-in image sensor on a smartphone. In addition, the broadband light source can be replaced with a cost efficient micro-lamp or light emitting diode and included with the spectrometer to make a complete spectrophotometer system, resulting in a compact DRS system suitable for POC applications. The DRS retrieval errors may be further decreased by improving the wavelength resolution of the smartphone spectrometer, which can be achieved by reducing the slit size, utilizing a G-Fresnel with finer grating period, and optimizing the optical alignment. Theoretical analysis previously indicates that 1 nm resolution can be achieved with a miniature G-Fresnel^33^.

We note that the use of tissue phantoms to estimate the performance of the smartphone spectrometer is subject to several limitations. Firstly, the liquid phantoms are treated as a homogeneous medium, which may not be an ideal model for heterogeneous or layered biological tissues. The DRS only measures volume-averaged absorber concentrations and scattering properties in tissues. It is challenging to quantify non-uniformities at high resolution. Secondly, the blood of *in vivo* tissues generally includes both oxy- and deoxy-hemoglobin molecules; but the hemoglobin molecules in the liquid phantoms used in the current experiments are mostly saturated with oxygen, which cannot be used to determine the accuracy for tissue oxygenation measurement. Finally, in the visible to near infrared wavelength range major scatterers are cell nuclei, mitochondria and collagen fibres with a size ranging from 10 micrometers to sub-micrometers. Therefore, future *in vivo* studies are necessary in order to fully evaluate the performance of the smartphone spectrometer for quantifying tissue hemoglobin contents.

## Conclusion

In this work, we demonstrated a G-Fresnel spectrometer that can be attached to a smartphone, and based on it a DRS system for quantitative diffuse reflectance measurement. The G-Fresnel spectrometer offers a compact package yet maintains ~ 5 nm wavelength resolution across the visible and the near-infrared region. Further, we have conducted measurement of the hemoglobin concentrations and reduced scattering coefficients of tissue phantoms using the smartphone spectrometer based DRS system. In comparison to a benchtop spectrometer based DRS system, our system demonstrates the feasibility to perform quantitative hemoglobin measurements with a comparable retrieval error of 9.2%. By virtue of the compact size, portability, and low cost of the demonstrated system, the efficacy of optical spectroscopy for quantitative measurement, and the ease of data collection, management, and computing afforded by smartphone technology, new POC opportunities with broad accessibility could thus be enabled.
